# Individualization of Piperacillin Dosing for Critically Ill Patients: Dosing Software To Optimize Antimicrobial Therapy

**DOI:** 10.1128/AAC.02664-14

**Published:** 2014-07

**Authors:** T. W. Felton, J. A. Roberts, T. P. Lodise, M. Van Guilder, E. Boselli, M. N. Neely, W. W. Hope

**Affiliations:** a The University of Manchester, Manchester Academic Health Science Centre, NIHR Clinical Research Facility in Respiratory Medicine, University Hospital of South Manchester NHS Foundation Trust, Manchester, United Kingdom; b Burns Trauma and Critical Care Research Centre, The University of Queensland, Brisbane and Royal Brisbane and Women's Hospital Brisbane, Queensland, Australia; c Albany College of Pharmacy and Health Sciences, Albany, New York, USA; d Laboratory of Applied Pharmacokinetics, University of Southern California, School of Medicine, Los Angeles, California, USA; e Department of Anaesthesiology and Intensive Care, Édouard Herriot Hospital, Hospices Civils de Lyon, Lyon, France; f Department of Molecular and Clinical Pharmacology, University of Liverpool, Liverpool, United Kingdom

## Abstract

Piperacillin-tazobactam is frequently used for empirical and targeted therapy of infections in critically ill patients. Considerable pharmacokinetic (PK) variability is observed in critically ill patients. By estimating an individual's PK, dosage optimization Bayesian estimation techniques can be used to calculate the appropriate piperacillin regimen to achieve desired drug exposure targets. The aim of this study was to establish a population PK model for piperacillin in critically ill patients and then analyze the performance of the model in the dose optimization software program BestDose. Linear, with estimated creatinine clearance and weight as covariates, Michaelis-Menten (MM) and parallel linear/MM structural models were fitted to the data from 146 critically ill patients with nosocomial infection. Piperacillin concentrations measured in the first dosing interval, from each of 8 additional individuals, combined with the population model were embedded into the dose optimization software. The impact of the number of observations was assessed. Precision was assessed by (i) the predicted piperacillin dosage and by (ii) linear regression of the observed-versus-predicted piperacillin concentrations from the second 24 h of treatment. We found that a linear clearance model with creatinine clearance and weight as covariates for drug clearance and volume of distribution, respectively, best described the observed data. When there were at least two observed piperacillin concentrations, the dose optimization software predicted a mean piperacillin dosage of 4.02 g in the 8 patients administered piperacillin doses of 4.00 g. Linear regression of the observed-versus-predicted piperacillin concentrations for 8 individuals after 24 h of piperacillin dosing demonstrated an *r*^2^ of >0.89. In conclusion, for most critically ill patients, individualized piperacillin regimens delivering a target serum piperacillin concentration is achievable. Further validation of the dosage optimization software in a clinical trial is required.

## INTRODUCTION

Infection in critically ill patients is associated with excessive morbidity, mortality, length of hospital stay, and health care costs (1, 2). Early and appropriate antimicrobial therapy is associated with improved clinical outcomes in a variety of clinical contexts (3, 4). Many licensed antimicrobial regimens are derived from studies in non-critically ill patients, and when they are applied to patients in the intensive care unit (ICU), suboptimal drug exposure may be the result for a significant proportion of critically ill patients (5, 6). Marked pharmacokinetic (PK) variability is characteristic of critically ill patients and may result from alterations in cardiac output, tissue perfusion, end-organ dysfunction, increased capillary permeability, hypoalbuminemia, and use of extracorporeal circuits. This pharmacokinetic variability results in a wide range of drug exposures (7). Low drug exposures increase the probability of clinical failure and emergence of antimicrobial resistance, while high drug exposures are associated with an increased likelihood of toxicity (8).

Piperacillin-tazobactam is widely used to treat infections in critically ill patients (9). For β-lactam agents, the fraction of the dosing interval that free drug concentrations are above the MIC (*fT*_>MIC_) is the pharmacodynamic index that best links drug exposure with the antibacterial effect (10). For piperacillin-tazobactam, the *fT*_>MIC_ of 50% of the dosing interval (50% *fT*_>MIC_) is associated with favorable clinical outcomes (8). The use of extended (or continuous) infusions of β-lactam antibiotics increases the *fT*_>MIC_ and may potentially improve overall efficacy (6, 11). Nevertheless, as many as 20% of patients receiving such regimens may still have suboptimal drug exposure (12). The exposure-response relationships determining emergence of antimicrobial resistance and occurrence of adverse events are less well defined. Trough (predose or minimum concentration of drug in serum [*C*_min_]) total β-lactam concentrations to MIC ratio of between 3 and 10 have been shown to prevent the emergence of antimicrobial resistance in dynamic *in vitro* infection models (13, 14). Piperacillin-tazobactam is usually well tolerated, and adverse events are typically detected in <2% of patients, but toxicity has been reported in as many as ∼50% of patients in specific cohorts (15). Dosage adjustment of antimicrobial regimens allows delivery of optimal drug exposures, allowing for variability in pharmacokinetics, aiming to maximize clinical efficacy, reduce the chance of adverse events, and suppress the emergence of antimicrobial resistance.

Therapeutic drug monitoring (TDM) is a standard of care for some antimicrobial agents such as gentamicin, vancomycin, and voriconazole (16–18). Increasing evidence suggests that TDM of β-lactam antibiotics (including piperacillin-tazobactam) may improve clinical outcomes in critically ill patients (19). Dose adaptation using Bayesian approaches offers a potential way of individualizing regimens for critically ill patients. This approach estimates a patient's (Bayesian posterior) pharmacokinetics using a combination of measured drug concentrations and information about the drug gained from previous experiences with that drug (quantified using population pharmacokinetic analysis). Dose-optimizing software can then identify the optimal dosage to achieve a predefined target drug concentration.

The aim of this study was to develop a population pharmacokinetic model to describe a large data set of critically ill patients. The population pharmacokinetic model was incorporated into the Bayesian dose optimization software program BestDose. We then assessed the accuracy of the dose optimization software using *in silico* validation experiments using multidose pharmacokinetic data from a small cohort of patients not included in the original population pharmacokinetic model.

## MATERIALS AND METHODS

**FIG 1:**
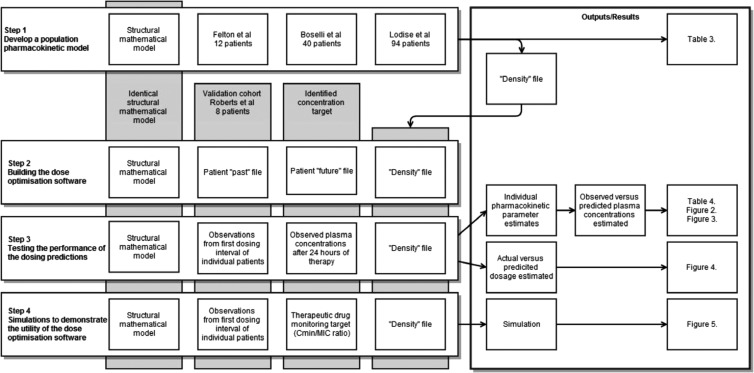
Overview of the development of the population pharmacokinetic model (step 1) and the building (step 2), testing (step 3), and demonstration (step 4) of the dosage optimization software. Pharmacokinetic data from 146 patients from three previous studies, Felton et al. (6), Boselli et al. (21), and Lodise et al. (20), were used. The validation cohort of Roberts et al. (27) was used.

An overview of the development of the population pharmacokinetic model and the building, testing, and demonstration of the dosage optimization software is shown in Fig. 1.

### Pharmacokinetic studies of critically ill patients.

Pharmacokinetic data from 146 patients from three previously published studies were obtained (6, 20, 21). A total of 803 piperacillin concentrations were available with each patient contributing 2 to 10 observations. Patients were administered between 2 and 4 g of piperacillin by 30-min infusion every 8 h, 4-h infusion every 8 h, or continuous infusion.

Lodise et al. provided data from 76 patients undergoing abdominal or thoracic surgery who received 2 g of piperacillin over 30 min (20). Plasma drug concentrations were measured in the first dosing interval. A further 18 patients undergoing colorectal surgery who had received 4 g of piperacillin over 30 min every 6 h were included. Plasma samples were obtained in the first and second dosing interval.

Plasma drug concentrations from a further 12 hospitalized patients, who had received 3 g of piperacillin over 4 h every 8 h were included (6). These patients were predominantly sampled on day 3 of therapy.

Finally, 40 ventilated patients with ventilator-associated pneumonia, administered piperacillin (12 g or 16 g) by continuous infusion, were included (21). Plasma samples were collected during the second 24 h of therapy. Weight and estimated creatinine clearance by Cockcroft-Gault were known for each patient (22). Piperacillin concentration was measured, in all three studies, using well-validated high-performance liquid chromatography assays.

### Development of a population pharmacokinetic model of piperacillin.

Data were analyzed using a population pharmacokinetic (PK) methodology using the nonparametric adaptive grid program Pmetrics 1.1.1 (23). Since no estimates of uniform assay error were available from the original studies, we utilized an assay error polynomial where standard deviation (SD) = 1.04 + (0.14 × C), where C is the drug concentration (in mg/liter). The polynomial was estimated by linear regression of the means and associated standard deviation for each of the three piperacillin concentrations for the 40 patients administered piperacillin by continuous infusion by Boselli et al. (21). In the fitting process, each concentration was weighted by its Fisher information, which is the inverse of the variance. Additionally, we chose the option in Pmetrics to multiply the variance by gamma (γ), which is an adaptive scalar that captures additional process noise such as errors in timing of samples or doses (23).

**FIG 2:**
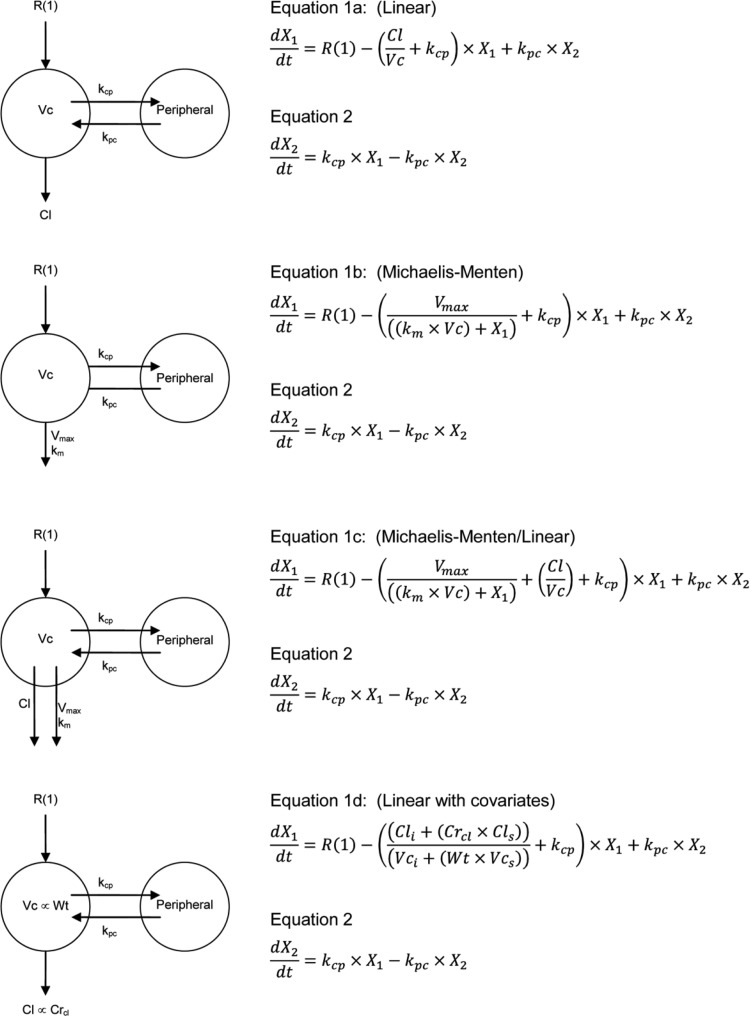
Structural mathematical models and associated differential equations. *X*_1_ and *X*_2_ are the amounts of piperacillin (in milligrams) in the central and peripheral compartments, respectively. *R*(1) represents the infusion of piperacillin. Cl (in liters per hour) is the clearance, and *Vc* is the volume of the central compartment (in liters). *V*_max_ is the maximum rate of clearance by the Michaelis-Menten clearance mechanism (in milligrams per hour), and *K_m_* is the concentration of piperacillin where clearance by the Michaelis-Menten clearance mechanism is half maximal (in milligrams per liter). *k*_cp_ and *k*_pc_ are the first-order intercompartmental rate constants. Cl_*s*_, fraction of piperacillin clearance due to creatinine clearance (in liters per hour); Cl_*i*_, clearance due to nonrenal means (in liters per hour); *V_i_*, volume of the central compartment not related to body mass (in liters); *V_s_*, volume of the central compartment proportional to body mass (in liters).

We evaluated several structural PK models. The details of these structural models are shown in Fig. 2. The models differed in the way in which piperacillin was cleared from the central compartment and by the covariates that were included. The models were parameterized in the following ways: (i) elimination by a first-order process (Fig. 2, equation 1a), (ii) elimination by a Michaelis-Menten process (Fig. 2, equation 1b), (iii) elimination by parallel first-order and Michaelis-Menten processes (Fig. 2, equation 1c), and (iv) elimination by a first-order process with creatinine clearance as a covariate and body mass as a covariate for the volume of the central compartment (Fig. 2, equation 1d). Piperacillin elimination, by a Michaelis-Menten process, is biologically plausible and has previously been identified to best describe the observed data in a population pharmacokinetic analysis (6).

For an individual patient without data, i.e., prior to any measured piperacillin concentrations, his/her parameter value joint distribution is the same as for the population. However, if observed data are available, the population distribution (i.e., the Bayesian prior) may be updated to a new distribution (i.e., the Bayesian posterior) that better predicts the individual's observations. The support points do not move, but their relative probabilities change, based on the ability to predict the patient's observed concentrations for his/her dosing history.

For each model, the final-cycle population parameter value distributions are summarized in terms of measures of central tendency (e.g., means and medians) and dispersion (e.g., standard deviation). Scatterplots of the observed-versus-predicted values for the population (i.e., Bayesian prior) and individual patients (i.e., Bayesian posterior) were examined. The fit of each of the four structural models to the data was assessed using a combination of the following: (i) the log likelihood value, (ii) the Akaike information criterion (AIC), and (iii) the coefficients of determination (*r*^*2*^) from a linear regression of the observed-predicted plots before and after the Bayesian step. Differences between the various models were also assessed statistically by calculating the difference in log likelihood values and comparing this value to a χ^2^ distribution with the degrees of freedom equal to the difference in the number of parameters between each model.

### Building the piperacillin dose optimization software.

We used the dose optimization software program BestDose to estimate each individual patient's pharmacokinetics and the optimal individual dosages for each patient. BestDose is based on software originally developed nearly 20 years ago by the Laboratory of Applied Pharmacokinetics, University of Southern California, Los Angeles (24). This dose optimization software has previously been used to individualize therapy with vancomycin, voriconazole, and antiretroviral therapy (25, 26).

The BestDose software requires four specific components: (i) a structural mathematical model that best describes the pharmacokinetics (we used the fourth structural pharmacokinetic model above; see Results); (ii) the “density” file (one of the outputs of the Pmetrics analysis), which serves as the Bayesian prior; (iii) a patient “past” file that contains the observed drug concentrations and details of the administered regimen; and (iv) a patient “future” file which contains the target drug concentrations deemed to be appropriate for that patient and initial estimates of the required drug dosages and frequency of administration.

The dose optimization software uses the equations in the model file and the population Bayesian prior in the density file, together with the individual patient's observed drug concentrations in the past data file to calculate a Bayesian posterior parameter value distribution for that patient. The dose optimization software then calculates the drug dose that minimizes the expected weighted squared error (over the Bayesian posterior distribution) between the predicted and user-specified target drug concentrations in the future data file.

### Testing the performance of the piperacillin dosing predictions.

A separate external data set from Roberts et al. (27) was then used to test the performance of the piperacillin dose optimization software. Piperacillin concentrations obtained from eight patients receiving 4 g of piperacillin as a 20-min infusion every 6 h (*n* = 7) or 8 h (*n* = 1) were used to validate the performance of the dose optimization software. Patients had a mean of 14 observations (range, 12 to 21) during the first dosing interval and a further 9 observations (range, 6 to 12) during a dosing interval 24 h later. The creatinine clearance was calculated for each patient using the Cockcroft-Gault equation.

Piperacillin concentrations collected during the first dosing interval were entered into the dose optimization software. To determine the optimal number of observations required, a comparison was made of one, two, three, or six observations. Piperacillin concentrations collected approximately 6 h (1 observation), 0.5 and 6 h (2 observations), 0.5, 3, and 6 h (3 observations), or 0.5, 1, 2, 3, 4, and 6 h (6 observations) after the start of the first dosage were utilized to describe each individual patient's pharmacokinetics. Using 1, 2, or 3 observations was investigated, as these are reasonable for routine clinical care, while 6 observations is sufficient to optimally estimate each of the pharmacokinetic parameters in a 6-parameter model. Each patient was administered 4 g piperacillin over either 20 or 40 min. The dose optimization software “past” data file contained the observed first dosing interval piperacillin concentrations for each of the eight individual validation patients. The “future” data file contained the required timings of the future dosages, an initial guess at the likely future dose that would be required to achieve desired concentration targets (in this case 4 g), the required piperacillin target concentration, and the timing of the target. For this *in silico* experiment, the target piperacillin concentrations at 24.5, 25, 26, 27, 28, and 30 h (6 observations) after the start of the first dosage were used as the observed piperacillin values after the first piperacillin dose on the second day of piperacillin dosing. The output from the dose optimization software was an estimate of (i) the individual's (post-Bayesian) pharmacokinetic parameters and (ii) the dosage required to achieve the observed piperacillin concentrations.

The ability of the dose optimization software was tested in two ways. First, the capability of the software to estimate each individual's pharmacokinetics was assessed by comparison of the observed-versus-predicted piperacillin concentration after 24 h of therapy. Simulations, utilizing parameter estimates from each of the eight validation individuals, was performed using ADAPT 5 (28). The piperacillin concentrations at the corresponding time points to the observed data during the second 24 h of treatment were estimated. The ability of the dose optimization software to predict the observed data was evaluated by the following: (i) visual inspection of the simulated piperacillin concentration-time profiles for the eight individual patients, including the observed piperacillin concentrations; (ii) linear regression of the observed-versus-predicted piperacillin concentrations for each of the eight validation individuals; and (iii) linear regression and estimation of the mean weighted prediction error (bias) and the bias-adjusted squared prediction error (precision) of the observed-versus-predicted piperacillin concentrations for all eight patients combined.

Second, the dose optimization software was tested by comparing the estimated delivered piperacillin dosage predicted from the observed piperacillin concentrations after 24 h of therapy. Assessment was made by comparison of actual and predicted piperacillin dosages after 24 h of treatment.

Linear regression was performed using GraphPad Prism version 5 for Windows (GraphPad Software, San Diego, CA, USA). Estimation of the mean weighted prediction error and the bias-adjusted squared prediction error was performed in R 3.0.1 (29).

### Simulations to demonstrate the utility of the dose optimization software.

The dose optimization software was finally used to predict the required dosage to achieve a predetermined piperacillin concentration. In this analysis, the same six time points, from the first 24 h of piperacillin therapy administered to the eight validation patients, were used. For each of the eight individuals, three doses of drug were administered in the first 24 h at 8-h intervals. This was to simulate the time required to measure the drug concentration after the first dose. Dosage adjustment was then performed for the fourth to seventh doses because day 2 of treatment represents an early opportunity for TDM intervention. The piperacillin target concentration was a predose (trough) concentration estimated from an *in vitro* hollow-fiber infection model, containing Pseudomonas aeruginosa. The identified concentration is associated with suppression of emergence of piperacillin resistance (30). The target trough total plasma piperacillin concentrations used were 13.6 mg/liter and 41.6 mg/liter for 30-min and 4-h infusion regimens, respectively. These concentrations would be applicable to a strain of Pseudomonas aeruginosa with a MIC of 4 mg/liter as used in the *in vitro* model.

## RESULTS

### Population pharmacokinetics of piperacillin in critically ill patients.

The demographics and clinical characteristics of the 146 patients used in this study are summarized in Table 1. A comparison of the fit of each of the four structural mathematical models to the data is shown in Table 2. All four models performed well. Evaluation of the log likelihood values against a χ^2^ distribution and comparison of Akaike information criterion indicated that the Michaelis-Menten model was superior to the other mathematical models. Examination of the linear regression of the observed-versus-predicted plots revealed that each model showed an *r*^2^ of >0.9, but the linear clearance model with covariates had a *y*-intercept value closest to zero. As all the models perform similarly, the linear clearance model with covariates, the most clinically relevant model, was used to predict the pharmacokinetics of critically ill individual patients and their optimal piperacillin dosage despite being statistically inferior to the Michaelis-Menten model. The mean, median, and standard deviation of the parameter estimates for the linear clearance with covariates population pharmacokinetic model are shown in Table 3.

**TABLE 1:** Demographics and clinical characteristics of the 146 patients used in this study

Demographic or clinical characteristic	Mean (median) [range] value for the characteristic in the following study^*a*^:				
	Felton et al.	Boselli et al.	Lodise et al.	Combined	Roberts et al.
Estimated creatinine clearance (ml/min)	115.0 (111.5) [38.0–169.1]	69.8 (52.0) [14.0–245.7]	89.0 (85.5) [27.0–221.0]	85.9 (81.0) [14.0–245.7]	165.5 (163.7) [58.8–256.6]
Wt (kg)	77.0 (71.5) [38.1–122.5]	73.0 (68.5) [49.0–113.0]	70.5 (69.0) [50.0–98.5]	71.7 (69.0) [38.1–122.5]	86.8 (82.5) [72.0–132.0]
Sex (no. of males:no. of females)	8:4	25:15	54:40	87:59	6:2
Age (yr)	46.8 (49.5) [20.0–69.0]	62.3 (64.0) [34.0–88.0]	54.2 (55.0) [18.0–78.0]	55.8 (56.0) [18.0–88.0]	43.6 (41) [19.0–75.0]
No. of patients	12	40	94	146	8
No. of doses	9.58 (9.00) [4.00–24.00]	48-h continuous infusion	1 or 2	N/A	4 or 5
No. of observations per patient	5.9 (6.0) [4.0–8.0]	3.0 (3.0) [3.0–3.0]	6.5 (6.0) [2.0–10.0]	5.5 (6.0) [2.0–10.0]	24 (24) [21–26]

a The values for three studies, Felton et al. (6), Boselli et al. (21), and Lodise et al. (20), are shown. The values for the three studies together are shown in the Combined column. The values for the validation cohort of Roberts et al. (27) are shown. N/A, not available.

**TABLE 2:** Evaluation of the predictive performance of piperacillin and tazobactam population models

Model	No. of variables	Log likelihood	χ^2^ compared to linear model	No. of cycles to convergence	AIC	Linear regression of observed-predicted values for each patient		
						*R*^2^	Intercept	Slope
Linear	4	−2,973.5	N/A	1,310	2,988	0.931	2.43	0.903
Michaelis-Menten	5	−2,892.5	2.26E−19	1,490	2,914	0.933	3.35	0.921
Parallel linear/MM	6	−2,894.5	7.00E−18	2,182	2,922	0.933	3.42	0.908
Linear with covariates^*a*^	6	−2,899.0	6.65E−17	1,061	2,930	0.925	2.27	0.918

a The model selected for the dose optimization software validation.

**TABLE 3:** Data on the parameter estimates for the linear clearance with covariates population pharmacokinetic model

Parameter^*a*^	Mean [median] ± SD
Cl*_i_*	3.83 [2.79] ± 3.35
Cl*_s_*	0.11 [0.10] ± 0.07
*V_i_*	4.54 [1.83] ± 4.43
*V_s_*	0.12 [0.06] ± 0.12
*k*_cp_	6.74 [0.85] ± 11.38
*k*_pc_	9.14 [1.65] ± 13.13

a Parameters defined in the legend to Fig. 2.

### Predicting pharmacokinetics of individual critically ill patients.

**FIG 3:**
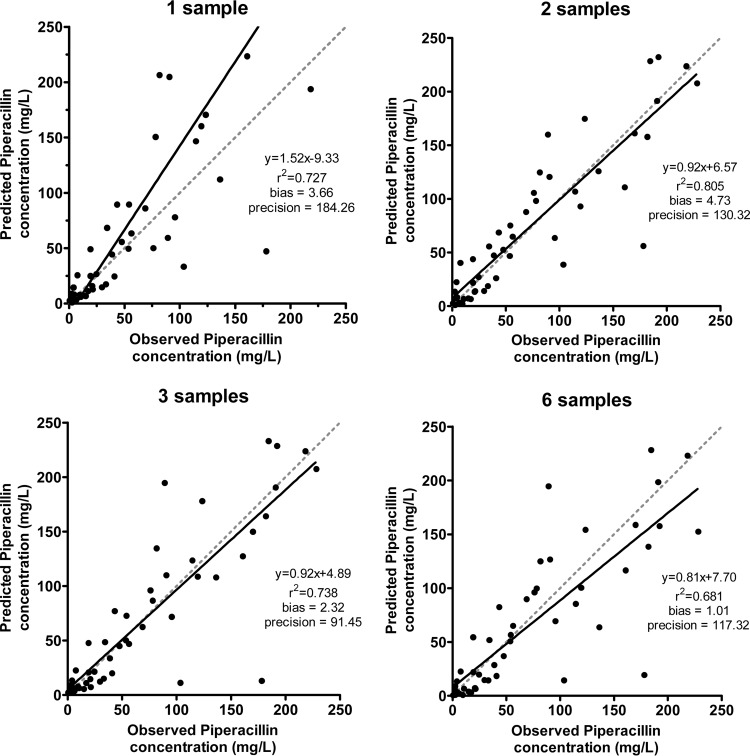
Observed-versus-predicted piperacillin concentrations for the *in silico* validation cohort using 1, 2, 3, or 6 measurements to determine the predicted postdose piperacillin concentrations after 24 h of therapy. The data points (●), linear regression (solid line), and unity (gray dashed line) are shown (bias is the mean weighted prediction error [mg/liter]; precision is the bias-adjusted squared prediction error [mg^2^/liter^2^]).

**FIG 4:**
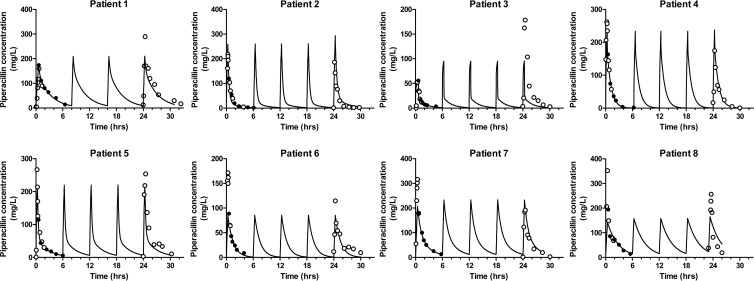
Piperacillin concentration-time profiles for eight validation patients generated from six observed piperacillin concentrations during the first dosing interval. Observed data entered into the software package (●) and observed data unknown to the software package (○) are shown. The predicted piperacillin concentration-time profiles are indicated by the solid lines.

Linear regression of the data for individual patients revealed *r*^*2*^ values ranging from 0.861 to 0.987 using a single observation, 0.893 to 0.987 using two observations, 0.883 to 0.979 for three observations, and 0.833 to 0.967 for six observations (Table 4). Linear regression of the combined observed-predicted values, from all eight individuals, revealed *r*^*2*^ values of 0.727 using a single observation, 0.805 using two observations, 0.738 using three observations, and 0.681 using six observations (Fig. 3). The mean weighted prediction error (bias) and bias-adjusted squared prediction error (precision) were 3.66 mg/liter and 184.26 mg^2^/liter^2^, respectively, using a single observation, 4.73 mg/liter and 130.32 mg^2^/liter^2^, respectively, using two observations, 2.32 mg/liter and 91.45 mg^2^/liter^2^ mg/liter, respectively, using three observations, and 1.01 and 117.32 mg^2^/liter^2^, respectively, using six observations. Visual inspection of the concentration-time profiles for each of the eight patients showed predicted concentrations were higher than the observed concentration following a single observation. For seven of the eight validation patients, utilizing more than one observation resulted in a satisfactory agreement between the observed piperacillin concentration at 24 h and the piperacillin concentration predicted by the population pharmacokinetic model (Fig. 4). For patient 3, the model did not predict the observed data well, but inspection of the raw data showed considerable differences between the observed piperacillin concentrations resulting from the first and fifth dosage.

**TABLE 4:** Linear regression of data for individual patients with different numbers of observations

Patient	1 observation		2 observations		3 observations		6 observations	
	*r*^2^	Predicted piperacillin dose (g) [% of delivered dose]	*r*^2^	Predicted piperacillin dose (g) [% of delivered dose]	*r*^2^	Predicted piperacillin dose (g) [% of delivered dose]	*r*^2^	Predicted piperacillin dose (g) [% of delivered dose]
1	0.861	2.68 [−33.0]	0.918	5.22 [30.5]	0.972	4.87 [21.7]	0.940	4.97 [24.3]
2	0.928	1.76 [−55.9]	0.947	1.15 [−71.3]	0.897	1.71 [−57.2]	0.897	1.67 [−58.2]
3	0.976	9.49 [137.3]	0.981	8.72 [118.1]	0.883	11.64 [191.0]	0.959	17.65 [341.2]
4	0.925	3.10 [−22.4]	0.919	3.05 [−23.9]	0.890	3.53 [−11.8]	0.833	3.42 [−14.5]
5	0.987	4.81 [20.3]	0.987	4.33 [8.3]	0.979	5.05 [26.1]	0.892	5.62 [40.5]
6	0.963	3.04 [−23.9]	0.920	3.83 [−4.3]	0.970	3.76 [−5.9]	0.938	5.05 [26.2]
7	0.967	1.85 [−53.8]	0.959	3.10 [−22.4]	0.960	3.32 [−17.0]	0.967	3.08 [−23.0]
8	0.905	1.89 [−52.6]	0.893	2.76 [−31.0]	0.893	2.54 [−36.4]	0.859	2.71 [−32.3]
Mean		3.58 [−10.5]		4.02 [0.5]		4.55 [13.8]		5.52 [38.0]
Median		2.86 [−28.5]		3.47 [−13.4]		3.65 [−8.8]		4.20 [4.9]

### Predicting the piperacillin dosage delivered to individual critically patients.

**FIG 5:**
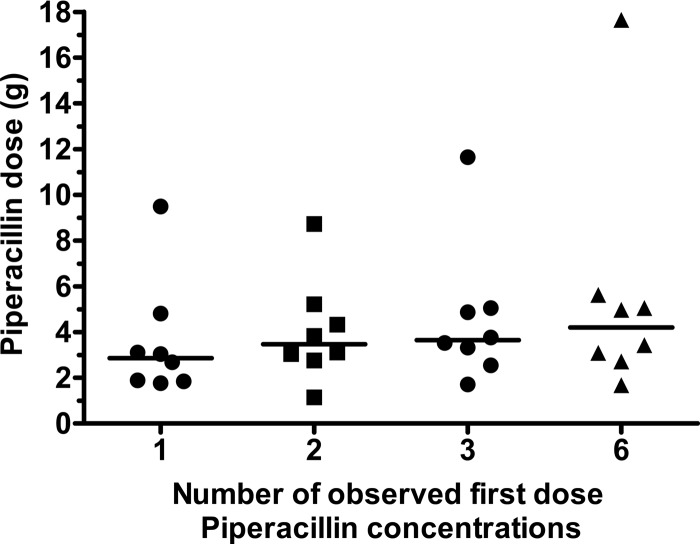
Comparison of the impact of entering one, two, three, or six known first-dose piperacillin concentrations into the dose optimization software on predicted piperacillin dosage for patients actually administered piperacillin (4 g). Each symbol represents the value for an individual patient, and the black lines represent the mean values for the groups of patients.

From a single postdose observation, collected following 24 h of therapy, the mean and median predicted piperacillin doses for the cohort of eight validation patients were 3.58 and 2.86 g, respectively, compared with a delivered dose of 4.00 g (Table 4 and Fig. 5). For two postdose observations, the mean and median predicted piperacillin doses were 4.02 and 3.47 g, respectively, compared to mean and median predicted piperacillin doses of 4.55 and 3.65 g following three observations and 5.52 and 4.20 g if six observations were used. When at least two observed piperacillin concentrations were used, the dose optimization software predicted that patient 3 required at least twice the administered dose of piperacillin to achieve the observed piperacillin concentrations. For this patient, inspection of the observed piperacillin concentrations (Fig. 4) shows that the piperacillin concentrations after 24 h of therapy are markedly higher than the piperacillin concentrations achieved after the first dose, but with little sign of significant drug accumulation.

### Examples of the clinical utility of the piperacillin dose optimization software.

**FIG 6:**
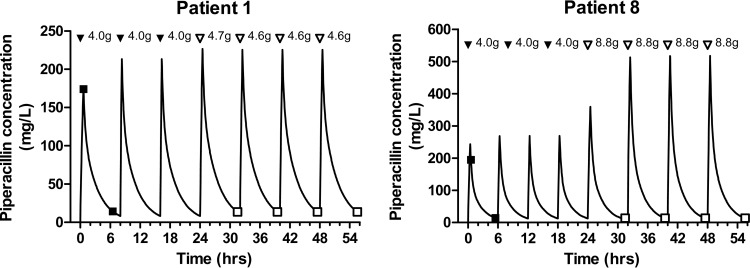
Predicted piperacillin concentration-time profiles for patients 1 and 8. The observed data entered into the software package (●) and the predicted piperacillin concentration (solid line) are shown. At the top of each graph, each arrowhead represents an administered dose (doses administered prior to individualization [all bolus doses] [▼] and doses administered following individualization [administration over 30 min for bolus doses] [▽]). The therapeutic drug target was a trough piperacillin concentration of 13.6 mg/liter.

The dose optimization software was used, *in silico*, to predict a specified trough piperacillin concentration following a 30-min administration of piperacillin every 8 h. In order to achieve the target piperacillin concentration, piperacillin 4.7 g and 8.8 g, administered every 8 h by 30-min infusion, were required for patients 1 and 8, respectively (Fig. 6).

## DISCUSSION

Infection remains a commonly encountered problem in critically ill patients and is associated with high morbidity, high mortality, and increased health care costs. Variation in the pharmacokinetics of β-lactam antibiotics occurs in critically ill patients (31). The use of a fixed regimen in critically ill patients will result in a wide range of drug exposures (32). Therapeutic drug monitoring (TDM) enables adjustment of the dose based on observed drug concentrations to achieve optimal drug exposure for an individual patient. TDM is a standard of care for some agents, such as gentamicin, vancomycin, and voriconazole, and is associated with improved clinical outcomes (16, 17). There is limited evidence suggesting that TDM improves achievement of pharmacodynamic targets for β-lactam antibiotics (19, 33).

Here, we develop and validate the necessary tools to enable dosage individualizuation of piperacillin in critically ill patients. Administration of β-lactams by extended or continuous infusion or through application of TDM has been suggested to exploit relatively detailed understanding of β-lactam pharmacodynamics. Continuous infusions have been shown to achieve the desired pharmacodynamic target in only ca. 80% of critically ill patients (12). This could, potentially, be improved using TDM and dose optimization. Typically, TDM is performed using dosing nomograms. However, the use of dosing nomograms in critically ill patients poorly predicts the required drug regimen (34, 35). This is due to the considerable pharmacokinetic variability in study populations. Additionally, dosing nomograms require assessment of a subject with the drug at pharmacokinetic steady state, whereas Bayesian dose optimization may be performed after the first dose. Finally, Bayesian dose optimization offers a truly personalized dosage for each patient rather than forcing the individual into one of several potential dosing bands. A pragmatic interpretation of the dosage identified by the dosing software may be required, so a practical and easy-to-administer dose could be prescribed to the patient. This dosage would result in a drug concentration that safely exceeds the identified plasma therapeutic target.

Both inter- and intrasubject pharmacokinetic variability may be important. Intersubject variability is fundamental to the argument for using TDM—i.e., inherent variability results in too many patients receiving suboptimal drug exposures. Intrasubject variability (due to the continually changing clinical state and pharmacokinetics observed in critically ill patients) has an impact on the ability to accurately predict a regimen that enables attainment of a desired therapeutic target in an optimally precise manner. This is illustrated by patient 3 in Fig. 4. Visual inspection of the concentration-time profiles (Fig. 4) illustrates the difference between the observed and predicted piperacillin concentrations after 24 h of therapy. This was due to a marked and rapid change in both piperacillin clearance and volume of distribution. Use of a mathematical nonlinear clearance model may have estimated the accumulation of piperacillin seen in patient 3. Additionally, optimal design of plasma sampling time points may maximize the information gained from each sample and allow better estimation of parameters, such as clearance and volume of distribution. It is likely that repeated assessment of a patient's observed drug concentration will be required, especially in patients with discernible changes in their clinical condition.

The change in pharmacokinetic parameters results from pathophysiological changes in critically ill patients. In order for a mathematical model to predict a patient's pharmacokinetic changes, a greater understanding of pathophysiological alterations is required. Quantification of these pathophysiological changes and subsequent incorporation as covariates into a mathematical model may allow better prediction of evolving pharmacokinetics and requires further study. Alternatively, a more pragmatic approach would be frequent observation and dosage adjustment performed with a minimum of delay between observation and adjustment. In this *in silico* validation, the time between the two sets of observations was 24 h. This is a reasonable estimate for the current amount of time it would take to process, measure, and then model the observed data before being able to change the regimen. Reducing the turnaround time would mean less time for pathophysiological alterations to change the dosing requirements. In unstable, critically ill patients, the process of measuring drug concentrations and establishing the optimal antimicrobial regimen may be a nearly continuous process. Reformatting drug assays to platforms, such as enzyme immunoassay, and away from chromatography may reduce time delays in producing drug concentration data. Additionally, moving the equipment required for TDM closer to the patient will minimize the turnaround time (36, 37). In the future, we anticipate that drug quantification will be performed in a similar way to glucose measurement using handheld devices at the bedside with built-in dosing software.

The drug exposure target used for dosage adjustment in the *in silico* validation experiment was identified in an *in vitro* dynamic pharmacokinetic/pharmacodynamic experiment (14). Here a strain of Pseudomonas aeruginosa with a MIC of 4 mg/liter was exposed to a range of piperacillin-tazobactam dosages. The identified target piperacillin concentration, expressed as a product of the MIC, was the lowest predose concentration required to suppress the emergence of piperacillin resistance. Two different TDM target concentrations were identified, both for piperacillin administered every 8 h, one by 30-min bolus injection and a separate target for 4-h extended infusions. To achieve this trough concentration, piperacillin dosages markedly higher than currently licensed may be required. This is illustrated by patient 8, who was predicted to require 7.4 g or 11.8 g of piperacillin, depending on the regimen, to achieve the target concentration. Use of these high piperacillin dosages would require attention to patient safety, although the resulting concentrations appear to be well tolerated (19).

The results of this *in silico* validation experiment illustrate the complexities of optimizing treatment for critically ill patients. The dose optimization software allows precise targeting of drug concentrations as previously demonstrated with voriconazole (38). The changing pathophysiology of critically ill patients and variability in pharmacokinetics makes delivery of optimized regimens challenging. Despite these challenges, the dose optimization software, when at least two observed concentrations were used, was able to provide a satisfactory response in seven of the eight patients. Rapid, near-patient drug quantification would reduce the impact of pharmacokinetic variability and may be required to further personalize and optimize antimicrobial therapies in the ICU. The ability of TDM and Bayesian dose adaptation of β-lactam antibiotics to improve outcomes in critically ill patients requires evaluation in prospective randomized studies.
